# Mechanism of Post-stroke Axonal Outgrowth and Functional Recovery

**DOI:** 10.14789/jmj.JMJ23-0025-R

**Published:** 2023-10-19

**Authors:** YUJI UENO

**Affiliations:** 1 Department of Neurology, University of Yamanashi, Yamanashi, Japan; 1 Department of Neurology, University of Yamanashi, Yamanashi, Japan; 2 Department of Neurology, Juntendo University School of Medicine, Tokyo, Japan; 2 Department of Neurology, Juntendo University School of Medicine, Tokyo, Japan

**Keywords:** stroke, axonal outgrowth, semaphorin 3A, exosomes, functional recovery

## Abstract

Axonal outgrowth after stroke plays an important role in tissue repair and is critical for functional recovery. In the peri-infarct area of a rat middle cerebral artery occlusion model, we found that the axons and dendrites that had fallen off in the acute phase of stroke (7 days) were regenerated in the chronic phase of stroke (56 days). *In vitro*, we showed that phosphatase tensin homolog deleted on chromosome 10/Akt/Glycogen synthase kinase 3β signaling is implicated in postischemic axonal regeneration. In a rat model of chronic cerebral hypoperfusion, oral administration of L-carnitine induced axonal and oligodendrocyte regeneration in the cerebral white matter, resulting in myelin thickening, and it improved cognitive impairment in rats with chronic cerebral ischemia. Recently, it has been shown that exosomes enhanced functional recovery after stroke. Exosome treatment has less tumorigenicity, does not occlude the microvascular system, has low immunogenicity, and does not require a host immune response compared to conventional cell therapy. Several studies demonstrated specific microRNA in exosomes, which regulated signaling pathways related to neurogenesis after stroke. Collectively, there are various mechanisms of axonal regeneration and functional recovery after stroke, and it is expected that new therapeutic agents for stroke with the aim of axonal regeneration will be developed and used in real-world clinical practice in the future.

## Introduction

Stroke is the leading cause of disability worldwide^1)^. In Japan, about 1.2 million people had strokes, and it is the fourth leading cause of death. In addition, medical expenses for stroke account for 11% in elderly persons, and they are expected to increase further in Japan, because Japan is facing a ‘super-aged' society.

Ischemic stroke accounts for about 80% of all ischemic stroke cases, and it has a variety of mechanisms^2, 3)^. In recent years, stroke medical care has made dramatic progress due to the spread of intravenous alteplase and intravascular thrombectomy for acute ischemic stroke, as well as the development of preventive medicine due to the emergence of various new antithrombotic drugs. However, once a stroke develops and severe disability occurs due to failure of such acute treatments, the burden on the patient and family is immeasurable. So far, sorts of agents have been tried and shown as effective for neuroprotection against ischemia in preclinical studies. However, these agents failed to show efficacy and safety in human stroke^4)^. Thus, there is an urgent need to develop alternative novel therapies that can facilitate functional recovery based on neuroregeneration.

Post-stroke axonal outgrowth is fundamentally related to recovery from functional impairment after stroke, and the several mechanisms have been elucidated^5, 6)^. In this paper, the results of our experimental research on axonal regeneration after stroke are reviewed.

## Molecular mechanisms including microRNA for axonal outgrowth

Peripheral sensory neurons activate a pro- regenerative program after nerve injury to enable axon regeneration and functional recovery. In contrast, regeneration of axons is poor in the central nervous system after injury. It is thought that there are various factors that inhibit axon regeneration, including Nogo, myelin-associated glycoprotein, oligodendrocyte-myelin glycoprotein, chondroitin sulfate proteoglycans present in injured scar tissue, and extracellular matrices such as semaphorin 3A^7, 8)^. On the other hand, there is an endogenous cAMP-mediated signal that regenerates axons after injury in neurons^9, 10)^.

An increasing number of studies have found that microRNA is involved in axonal growth^11, 12)^. In superior cervical ganglia neurons, miR-338 locally regulates mitochondrial activity in axons^12)^. It was shown that attenuation of miR-9 in embryonic cortical neurons facilitated axonal outgrowth by targeting microtubule-associated protein 1b^11)^. The miR-17-92 cluster is a typical highly conserved polycistronic miRNA cluster, which is located in human chromosome 13, encoding six mature miRNAs: miR-17, miR-18a, miR-19a, miR-19b, miR-20a, and miR-92a^13)^. The miRNA-17-92 cluster may be highly expressed in a wide range of tumor cells and types of cancer, such as lung, breast, pancreatic, prostate, and thyroid cancers, and lymphomas^14)^. We focused on the miR-17-92 cluster and investigated whether the miR-17-92 cluster enhances axonal outgrowth in primary cultured neurons. We found that the miR-17-92 cluster was expressed in the distal axons of the neurons. Overexpression of the miR-17-92 cluster in cortical neurons significantly increased axonal outgrowth, whereas distal axonal attenuation of endogenous miR-19a suppressed axonal outgrowth. Overexpression of the miR-17-92 cluster reduced Phosphatase and Tensin Homolog Deleted from Chromosome 10 (PTEN) (PTEN) proteins and elevated phosphorylated mammalian target of rapamycin (mTOR) in the distal axons. In contrast, distal axonal attenuation of miR-19a increased PTEN and inactivated mTOR in the axons, but alterations of these proteins were not prominent in the cell bodies. Thus, we showed that axonal alteration of miR-17-92 cluster expression enhanced axonal outgrowth with regulation of PTEN/mTOR signaling^15)^.

## Post-stroke axonal-outgrowth in the peri-infarct area

Many stroke patients show some degree of functional recovery a few months after their stroke event, which is related to post-stroke axonal outgrowth. Axonal outgrowth has been studied in experimental stroke models. The stroke induces sprouting of axons from contralateral cortex into the ipsilateral red nucleus^16)^ and the ipsilateral cervical spinal cord^17, 18)^.

In the peri-infarct area, which is an area from the margin of the ischemic core to 300 μm from the ischemic core in the middle cerebral artery occlusion model (MCAO), there could be plasticity that induces reconstruction of the neural networks and brain repair after stroke injury^19, 20)^. It has been shown that ATRX and GDF10 are related to axonal regeneration in the peri-infarct area^5, 21)^. In our previous study, we evaluated axonal outgrowth in the peri-infarct area from the acute to chronic phases of ischemia^6)^. The expression of phosphorylated neurofilament heavy chain (pNFH), a marker for axons, decreased on the 7th day of MCAO, but it increased substantially to the chronic phase on the 28th and 56th days after MCAO. Moreover, pNFH^+^ axons were myelinated by oligodendrocytes. Regeneration of dendrites and dendritic spines was also observed 56 days after MCAO. In cultured cortical neurons, we analyzed the pNFH levels by western blotting after oxygen-glucose deprivation (OGD) for 3 h which was *in vitro* model of acute ischemic stroke. We found a substantial increase in pNFH levels 96 hours after OGD, which corresponds to the chronic stage of cerebral infarction, together with downregulation of PTEN and upregulation of phosphorylated Akt and phosphorylated glycogen synthase kinase 3*β* (GSK-3*β*). Administration of an Akt inhibitor after OGD resulted in decreased expression of pAkt, downstream pGSK-3*β*, and pNFH, whereas administration of a GSK-3 inhibitor decreased expression of pGSK-3*β* and increased expression of pNFH. In the peri-infarct area, pGSK-3*β*+ fibers were co-localized with pNFH+ fibers in a rat MCAO model. Collectively, we found that axonal outgrowth is regulated through PTEN/Akt/GSK-3*β* signaling after stroke^6)^.

Axonal navigation was facilitated by several guidance molecules with attractive and repulsive signals on their growth cones. Reactive astrocytes form glial scars that hinder axonal regeneration in the peri-infarct area^22)^. On the contrary, it was shown that glial scars are essential for axonal regeneration after spinal cord injury^23)^. Semaphorins are a large family of guidance cue proteins, and semaphorin 3A (Sema3A) is a secreted protein that has been shown to inhibit axonal growth. After spinal injuries and optic nerve axotomy, Sema3A is implicated in scar formation^8, 24)^. Sema3A is also expressed in ischemic neurons after stroke^25)^. A previous study showed that inhibition of Sema3A enhanced axonal regeneration and improved functional recovery after spinal cord injury^8)^. In Sema3A signaling, downstream of Sema3A through the NRP1/PlexA1 complex, Rnd1 and R-Ras are linked with Akt/GSK-3*β* signaling. Thus, we had sought to analyze whether inhibition of Sema3A regulated Rnd1/R-Ras/Akt/GSK-3*β* signaling and axonal outgrowth after ischemia. Using cortical neurons *in vitro*, the sema3A inhibitor downregulated Rnd1 and upregulated R-Ras, which in turn activated Akt and pGSK-3*β*, increasing pNFH after OGD. It was found that pGSK-3*β* was co-localized with pNFH axons. In a rat MCAO model, expression of Sema3A in neurons increased in the acute phase of stroke, reached a peak at 14 days, and then decreased to 56 days after MCAO. We administered a sema3A inhibitor into the peri-infarct area using an osmotic mini-pump. A high dose of the sema3A inhibitor significantly increased pNFH^+^ axons and neuronal GSK-3*β* in the peri-infarct cortex, and it enhanced functional recovery during the recovery period in a rat MCAO model^26)^.

## L-carnitine improves cerebral white matter injury and cognitive impairment

Ligation of bilateral common carotid arteries (LBCCA) induces chronic cerebral hypoperfusion, which results in cerebral white matter injury and cognitive impairment in rats, and is a model of vascular dementia. Administration of edaravone for three consecutive days after LBCCA upregulated eNOS levels in endothelial cells in the cerebral white matter and ameliorated white matter injury 28 days after LBCCA^27)^.

L-carnitine has potent antioxidant and anti-inflammatory effects, and it has been reported to improve intermittent claudication in patients with peripheral arterial disease and to promote myocardial remodeling in patients with acute myocardial infarction^28, 29)^. As for the therapeutic effect of L-carnitine on cerebral infarction, it has been reported that L-carnitine suppressed the loss of neurons due to its antioxidant effect in a rat model of transient cerebral ischemia^30)^. In our previous study, 600 mg/kg of L-carnitine daily were administered orally to rats subjected to LBCCA for 28 days. L-carnitine-treated rats showed a significant reduction of escape latency in the Morris water maze task 28 days after LBCCA. On western blot analysis using samples of corpus callosum, L-carnitine increased protein levels of pNFH, together with a reduction in phosphorylated PTEN, and it increased phosphorylated Akt and mammalian target of rapamycin (mTOR) 28 days after LBCCA. On immunohistochemistry, L-carnitine suppressed lipid peroxidation and oxidative DNA damage, and it enhanced oligodendrocyte marker expression and myelin sheath thickness after LBCCA. The regulation of the PTEN/Akt/mTOR signaling pathway by L-carnitine enhanced axonal plasticity while ameliorating oxidative stress and increasing oligodendrocyte myelination of axons. Thus, L-carnitine improved white matter lesions (WMLs) and cognitive impairment in a rat chronic hypoperfusion model^31)^.

## Exosomes as a therapy for stroke recovery

Exosomes, extracellular vesicles that are 40 to 100 nm in diameter, and enriched in microRNA, mRNA, nucleic acids, lipids, and proteins, exert intercellular communication in CNS not only under normal physiological conditions, but also under pathological conditions^32-34)^. Exosome treatment is superior to cell therapy because: (1) it has less tumorigenicity; (2) it does not occlude the microvascular system; (3) it has low immunogenicity not requiring a host immune response; and (4) tough lipid bilayer vesicles retain bioactivity^34-37)^. Treatment with exosomes has been proven to be a good candidate for not only myocardial injury, but also kidney injury, by suppressing the inflammatory reaction and oxidative stress, and enhancing repair of injured tissues^38-43)^.

In the central nervous system, exosomes exert important roles in cell-cell communication in brain remodeling after stroke^34)^. It has been shown that exosomes derived from mesenchymal stromal cells (MSCs) in stroke treatment displayed the same tissue regeneration capability as MSCs themselves, and other studies demonstrated that treatment with such exosomes enhanced not only neurogenesis, angiogenesis, and axonal outgrowth, but also suppression of inflammatory reactions^44-47)^. It was demonstrated that prion proteins in astrocyte-derived exosomes increased after ischemia, which exerted neuroprotection *in vitro*^48)^. Xin et al showed that administration of MSC-derived exosomes with high enrichment of miR-133b facilitated the release of astrocyte-derived exosomes, which increased neurite outgrowth^49)^. Polarization of microglia induced by IL-4 increased miR-26a in microglia-generated exosomes, which may promote tube formation *in vitro* and angiogenesis *in vivo* after ischemia^50)^. In our previous study, inhibition of Sema3A in ischemic astrocytes downregulated miR-30c-2-3p and miR-326-5p in astrocyte-derived exosomes, which had the capability of promoting axonal elongation in ischemic neurons with upregulation of prostaglandin D2 synthase^32)^.

## Conclusions

There is an urgent need to develop novel therapies to enhance functional recovery based on axonal outgrowth. The inhibition of inhibitory molecules for axonal outgrowth and exosomes can be a new therapeutic candidate for stroke recovery.

## Funding

This work was supported in part by the Japan Society for the Promotion of Science (grant numbers 17K09764 and 20K07909 to Y.U.), Japan Science and Technology Agency FOREST (Grant No. JPMJFR210K), a Grant-in-Aid (S1411066) from the Foundation of Strategic Research Projects in Private Universities from the Ministry of Education, Culture, Sports, Science, and Technology, a grant from the Suzuken Memorial Foundation, and a grant from the Takeda Science Foundation.

## Author contributions

YU-Drafting the manuscript, study concept, acquisition of data, supervision and coordination, and reading and approving the final manuscript.

## Conflicts of interest statement

The author declares that there are no conflicts of interest.
